# A Handbook of Physical Diagnosis

**Published:** 1880-05

**Authors:** 


VI.—A Handbook of Physical Diagnosis. By Dr. Paul Guttman, Private Docent in Medicine, University of Berlin. Translated from the third German edition by Alex. Napier, M.D., Fel. Fac Phys. and Surg., Glasgow, with a colored plate and eighty-nine fine wood cut engravings New York. Wm. Wood & Co.

These books are of the very finest mechanical execution, and treat in a proper manner very important practical subjects. Each volume has over 350 pages, and are therefore very cheap standard works.

In addition to the four volumes received, the series for 1880 will consist of A Treatise on the Continued and Periodical Fevers, by James C. Wilson, M.D.; Diagnosis and Treatment of Diseases of the Ear, by Albert H. Buck, M.D.; Therapeutics, translated by D. F. Lincoln, M D., from the Materia Medica and Therapeutics of A. Trosseau, M.D. in three volumes; A Treatise on common forms of Functional Nervous Diseases, by L. Putzel, M.D.; Minor Surgical Gynecology, a manual of practical hints and methods of procedure in gynecological practice, for the use of practitioners in medicine, by Paul F. Munde, M.D.; and A Practical Manual of Diseases and Deformities of the Joints, with special reference to their diagnosis and mechanical treatment, particularly designed for the use of general practitioners of medicine, by LeRoy Milton Yale, A.M., M.D.

